# Supplementary material for: Can the creation of new freshwater habitat demographically offset losses of Pacific salmon from chronic anthropogenic mortality?
Source: PLoS One. 2020 Dec 17;15(12):e0237052. doi: 10.1371/journal.pone.0237052 (PMC7746168; doi:10.1371/journal.pone.0237052)
Supplement: S1 File — (DOCX) [file pone.0237052.s001.docx]

##Example of R code for population model (Gibeau et al, PONE-D-20-21844)

## Initial population size

Nadult.0 <- 808

Nfry.0 <- 13379

Nsmolt.0 <- 5249

### Parameters

Feggs <- 2597

Pfem <- 0.452

Pem <- 0.223

a21= 0.392

a32=0.154

aBH=0.5

kBH= 15318

BH=function (Dfry, a, k)

{

(a*Dfry) / (1+(a/k)*Dfry)

}

num.years=45 ## 15 generations of 3 years life-cycle

num.iters=1

## S_dist = survival after disturbance, i.e. after the extra chronic mortality is applied

S_dist <- c(1, 0.98, 0.95, 0.93, 0.9, 0.85, 0.8)

## Example of model run

## Mean production of habitat compensation (0.47 smolts per m2)

## 2 % of chronic mortality

## mean number of smolts produced per size of habitat compensation (from 100m2 to 25,000m2)

Smolt_compH <- c(0, 47,235,470,705,940,1175,1410,1645,1880,2115,2350, 3525,4700,5875,7050,8225,9400,11750)

## Minimum number of adults spawning in the compensation habitat to produce the number of smolts above

# Corresponds to: number of smolts produced divided

## by (freshwater survival (0.75) x egg-to-fry survival (0.223) x Feggs x Pfem)

Na_compH<- c(0, 2,11,23,35,47,59,71,83,95,107,119,179,239,299,359,418,478,598)

## Example of simulation run for fry life-stage affected by chronic mortality ##

N.adult=list()

N.fry=list()

N.smolt=list()

for(k in 1:18){

N.fry[[k]] <- matrix(nrow=num.years, ncol=num.iters)

N.smolt[[k]]<- matrix(nrow=num.years, ncol=num.iters)

N.adult[[k]] <- matrix(nrow=num.years, ncol=num.iters)

}

for(k in 1:18) {

Pop <- data.frame(N.fry=rep(NA, num.years),N.smolt=rep(NA, num.years),N.adult=rep(NA, num.years))

Pop[1,] <- c(Nfry.0,Nsmolt.0, Nadult.0)

for(j in 1:num.iters) {

for(i in 2:num.years) {

F13a= Feggs * Pfem * Pem

a21= a21

a32=a32

Pop[i,1] = (BH(((Pop[i-1,3]-Na_compH[k]) * F13a), aBH, kBH)) *S_dist[2]

Pop[i,2] = (Pop[i-1,1]+ Smolt_compH[k]) * a21

Pop[i,3] = Pop[i-1,2]* a32

Pop[i,] = floor(Pop[i,])

N.fry[[k]][,j] <- Pop[,1]

N.smolt[[k]][,j] <- Pop[,2]

N.adult[[k]][,j] <- Pop[,3]

}

}

}

## This simulation was repeated for S_dist[i]= 1, 0.98, 0.95, 0.93, 0.9, 0.85, 0.8;

## and for varying production in compensation habitat (25%, median, mean, and 75% of smolts produced,

## i.e. 0.1, 0.18, 0.47, and 0.54 smolts per m2), which affects the parameters Na_compH and Smolt_compH

### Other scenarios:

## When eggs were affected by chronic mortality, parameter F13a changed to

F13a= Pfem *Feggs *S_dist[i] * Pem

## and line 58 changed to

Pop[i,1] = (BH(((Pop[i-1,3]-Na_compH[k]) * F13a), aBH, kBH))

## When smolts or adults were affected by chronic mortality, lines 58 and 59 changed to

Pop[i,1] = (BH(((Pop[i-1,3]-Na_compH[k]) * F13a), aBH_mean, kBH))

Pop[i,2] = (Pop[i-1,1]+ Smolt_compH[k]) *S_dist[i] * a21
