# Supplementary material for: Can the creation of new freshwater habitat demographically offset losses of Pacific salmon from chronic anthropogenic mortality?
Source: PLoS One. 2020 Dec 17;15(12):e0237052. doi: 10.1371/journal.pone.0237052 (PMC7746168; doi:10.1371/journal.pone.0237052)
Supplement: Attachment — Submitted filename: Review_PONE-D-20-21844.docx [file pone.0237052.s002.docx]

**Reviewer Comments: Can the creation of new freshwater habitat demographically offset losses of Pacific salmon from chronic anthropogenic mortality?**

This paper evaluates the efficacy of “out-of-kind” habitat creation in bolstering threatened coho salmon populations. The authors use a matrix population model with three life stages to demonstrate that the necessary amount of compensatory habitat may be much larger than what is currently included in many mitigation projects. The study is purely theoretical in its approach. I think this is the primary shortcoming of the manuscript as the authors have not developed a novel type of model or analytical technique, nor have they included any empirical data to validate their findings. Nevertheless, the study does attempt to fill a critical knowledge gap with respect to aquatic habitat restoration and management and is an interesting read overall. If the authors can conduct major revisions on the structure of their stage-structured population model and their justifications for using the parameter values and model scenarios that they did, I think this article will be suitable for publication in *PLOS One*.

**Abstract**

Lines 5-7: My suggestion would be to delete this sentence, as the theme of this paper is specifically centered on salmonid habitat loss/creation.

Line 11: “in combination” is unclear here. Do you mean in combination with each other? An example of how you might re-phrase this sentence is to say you “used a stage-structured population model where each life stage was evaluated individually and as a component of the full model.”

Line 17: Is widespread mortality at multiple life stages typical for coho salmon? Doesn’t it depend on the type of development action being enacted? How do mortality rates differ among life stages? I think there is room for a brief explanation of why coho salmon are an exemplary case study species (see below).

Lines 18-19: I had trouble deciphering this sentence (specifically: “than if we assumed mean productivity or as compared to the typical size built in the PNW”). Please clarify.

**Introduction**

The introduction section is generally well formulated but would benefit from some changes to organizational structure and content.

1) I recommend shortening and combining the first two paragraphs of the introduction section and adding more history about the use of mitigation actions specifically for salmon habitat. I understand the impetus for starting with such broad subject matter — the readers of PLOS ONE have varied backgrounds outside of salmon ecology. That being said, many of them will be less interested in the jargon and more interested in the authors’ justification for conducting such a study.

2) I understand that theoretical approaches do not always fall into the “hypothesis-based science” category, but it would be nice to see more justification for your approach. The last paragraph would be strengthened if gaps in knowledge, study objectives, and predictions were explicitly listed out.

3) Readers would benefit from more information about coho salmon life history strategies and why they are an exemplary species for evaluating the efficacy of out-of-kind mitigation projects. Similarly, it’s unclear why river and stream restoration projects are considered “out-of-kind” to begin with. More explanation about this would be beneficial.

Line 37 and throughout: Please move references to the end of the sentence whenever possible.

Line 41-42, 83, and throughout: I find that the way the terms “compensation,” or “compensation activities,” or “compensation habitat,” are used to read awkwardly. Is compensation habitat a policy term? If not, I would consider changing to “compensatory habitat” instead.

Line 45: Please change to “compensating and offsetting activities.” Also clarify, are the shortcomings characteristics of compensation and offsetting themselves or the metrics used to evaluate them? Both?

Lines 58-59 and throughout: Please reconsider the use of passive voice throughout your document. Using the active voice is often a more effective way of communicating your science and tends to read more easily. e.g., “developers proposed to compensate for fish entrainment by removing a dam that was 50-kilometers from the offending nuclear powerplant.”

Lines 60-62: Please clarify. Were the restoration efforts themselves examples of out-of-kind compensation or was it the effort to run the population model?

Lines 75-79: What about agricultural and urban development?

Line 82: It would seem that restoration is a very specific example of out-of-kind compensation. How does this exemplify this strategy as a whole? I would like to see more justification for 1) why river and stream restorations are examples of out-of-kind compensatory actions. It would seem to me that a lot of time restoration actions occur in areas directly adjacent to habitat loss or mortality-causing structures, and 2) why coho salmon are an optimal species to use in this kind of theoretical case study (see #3 above).

**Methods**

I haven’t worked with stage-structured population models in years, so I won’t speak to whether or not the authors have used them correctly here. What I would like to see is a table outlining each of the model simulations (e.g., egg mortality, fry mortality, adult mortality, multiple sources of mortality) and which parameters in the model were manipulated. I think readers would also appreciate an explicit ecological justification (with references) as to why and how certain parameters were manipulated for each simulation.

What does “productivity” mean in this context? Does it refer specifically to the additional number of eggs and fry produced each year due to restored and created spawning habitat, or does it also include potential benefits to juvenile salmon growth and survival in response to added prey resources? If it does *not* include the latter, I think this is a major shortcoming in the described analysis — that restored and created habitats contribute to reproductive productivity and little else. The model likely underestimates survival rates and population growth, as added habitat can bolster productivity not only by adding physical space for redds, but also nursery habitat with terrestrial and aquatic prey subsidies and shelter from predation*. I would like to see an effort to include these habitat benefits in the model, or at the very least in a detailed paragraph in the discussion section.* For example, you could modify the carrying capacity (*k*) value in the Beverton-Holt density dependence function based on the amount of simulated compensatory habitat.

Lines 98-101: For clarity, I would rewrite this sentence as “To evaluate the amount of compensatory habitat required to maintain productivity for coho salmon populations experiencing chronic mortality, we modelled the offsetting potential of constructed habitats for salmon eggs, parr, and smolts/adults.”

Line 99 and throughout: Note that “salmon” should always come after “coho.”

Lines 107-112: Please move this information to the last paragraph of the introduction section. The paragraph can then be started something like: “We used several model scenarios to…”

Line 121: I’m surprised about the grouping between smolts and adults, as they experience very different drivers of mortality during the smoltification/out-migration phase and upstream migration/return phase. Either the model should be structured such that smolts and adults are two separate life stages in the matrix, or the authors should provide better justification as to why these very distinct life stages are grouped together.

Line 162: Why was density dependence omitted from the baseline model? Isn’t this something that juvenile salmon encounter even if their habitat is pristine?

Line 162: I don’t see the initial population sizes listed anywhere.

Line 168: This assumes that adult salmon are actively choosing to spawn in constructed side channel habitat with priority over main channel habitat. I worry about the validity of this assumption and I think it requires greater justification.

Line 200: I would move this elsewhere so it’s not its own paragraph.

Line 210: Which citation was supposed to go here?

**Results**

Line 216: Wasn’t this specifically the fry stage where you applied the Beverton-Holt function? Please clarify.

Line 220: Was this supposed to be Fig. 2?

Lines 232 and 234: No need to reference a figure multiple times in the same paragraph.

Lines 237-246: It appears you’ve accidentally pasted the figure caption here.

Line 256: Please see the comment above. It’s unclear what you mean by “productivity.” I’m also confused about whether your model assumes juvenile salmon stay in the same reach in which they are born. A fry produced in constructed side channel habitat will not stay there, but will move throughout the watershed until it migrates to the sea. Likewise, fry reared in the mainstem river will take advantage of constructed side channels periodically to feed and avoid predation. At what point are these two habitats considered separate and at what point are they combined in the model?

Line 262: The importance of the fry-smolt survival parameter further highlights the value of modifying the carrying capacity (k) value in the Beverton-Holt density dependence equation to account for the added side channel habitat.

**Discussion and Conclusions**

Lines 270-273: Just a reminder that your model was run specifically for coho salmon and this limitation needs to be elaborated on to begin with.

Line 279: See comments above. How is productivity defined here?

Lines 284-288: I don’t see anywhere how you tested the effect of location on the magnitude of mitigation. I can see that you’re trying to substitute life stage for location here, but juvenile salmon can move throughout a watershed depending upon environmental conditions, prey availability, and ontogenetic cues, so the assumption that they would only occupy a habitat up or down stream of a created wetland needs to be further justified.

Line 295: I’m glad you mentioned this an I think it’s worth expanding upon. Size is not everything when it comes to bolstering salmon populations. Adults and juveniles require appropriate habitat features to bolster their reproduction, growth, and survival, such as pebbly bottom substrate, large woody debris for shelter from predators, and overhanging vegetation to support prey inputs. An agency could build many hectares of stream habitat, but if it is not structurally and functionally useful then it may have little effect on salmon populations at all.

Lines 296-298: Please be wary of confusing productivity with habitat quality. Productivity refers specifically to the actual quantity of fish produced in terms of number of spawn or physical growth (in g/day). Also, what does “more demographically efficient” mean?

The Conclusions section should be framed in broader terms as in lines 377-382. Avoid using phrases like “our results” or “our analysis” and try to focus more on the big picture.

**Other**

Please consider making your model code publicly available on GitHub or a similar repository and linking to it in a Data Availability Statement.
