# Supplementary material for: Can the creation of new freshwater habitat demographically offset losses of Pacific salmon from chronic anthropogenic mortality?
Source: PLoS One. 2020 Dec 17;15(12):e0237052. doi: 10.1371/journal.pone.0237052 (PMC7746168; doi:10.1371/journal.pone.0237052)
Supplement: Attachment — Submitted filename: Response to Reviewers 1.docx [file pone.0237052.s003.docx]

**Reviewer #1**: Review of “Can the creation of new freshwater habitat demographically offset losses of Pacific salmon from chronic anthropogenic mortality?”

Gibeau et al.


The authors tackle a very complex issue within the sphere of freshwater restoration and mitigation for anthropogenic impacts to these habitats. Development of these types of models are beneficial to managers who need data-driven tools to justify the size or scope of mitigation actions required to offset adverse impacts of human activities.
The scope of this analysis is more limited than expressed in the introduction and discussion. For this tool to be useful to managers, model inputs would be needed from local impacted populations. As is, coho productivity and offsetting habitat size inputs are from a number of streams in a relatively small geographic area-SW British Columbia and NW Washington-nothing evident from larger systems like the Fraser or Columbia Rivers. However, the authors do an excellent job of demonstrating the potential of such an analysis if the inputs are available. The results are clearly communicated along with a good discussion of how the results may differ given variation in productivity estimates or mortality impacting more than one life stage. The greatest weakness of this analysis is that it relies on knowing the magnitude of added mortality due to human impacts, which is difficult to predict for an impact that has yet to happen. The authors acknowledge this shortcoming in the closing sentences of the discussion but do not elaborate further. A deeper discussion of the limitations of the model should be included.
The authors state that data are available upon request, however, more information on the geographic nature of the data used in the model would be useful to the reader.
Overall, the paper is very well written and will certainly contribute to the literature on quantifying and compensating for human impacts to salmon species.

Thank you for the positive and helpful comments. We revised the introduction and discussion to better focus the language. We agree that the usefulness of the model for managers relies on the quality and availability of the parameters from local populations, and that the model can be easily adapted when and where such data are available. To that effect, we added a simplified example of our R code as a Supplemental Material, so that any scientist or manager can update the simulations to specific populations. As suggested, we also developed our discussion around the limitations represented by the uncertainty around specific mortality rates, and moved it earlier in the discussion to emphasis its importance (lines 313-39).

We would also like to clarify that all data and parameters needed to run the simulations are included in methods and in Table 1. We added more details in the methods about how we derived the various parameters, along with the reference papers, so that the geographic nature of the data is clearer and more accessible (lines 131-141).

We are confident that all these edits and modifications improve the manuscript and we thank you for the suggestions.

A few specific comments:
References to figures need to be double-checked.

*Yes, we double-checked the references to figures and adjusted them when needed.*

There is a large section where the caption for figure 2 has been incorporated into the text (lines 237-247).

*Thank you for noting that; we removed it.*

There is a missing citation on line 210.

*Thank you for noting that as well, we added the needed citation.*

**Reviewer #2**: My review is attached as a word document.

**Reviewer Comments: Can the creation of new freshwater habitat demographically offset losses of Pacific salmon from chronic anthropogenic mortality?**

This paper evaluates the efficacy of “out-of-kind” habitat creation in bolstering threatened coho salmon populations. The authors use a matrix population model with three life stages to demonstrate that the necessary amount of compensatory habitat may be much larger than what is currently included in many mitigation projects. The study is purely theoretical in its approach. I think this is the primary shortcoming of the manuscript as the authors have not developed a novel type of model or analytical technique, nor have they included any empirical data to validate their findings. Nevertheless, the study does attempt to fill a critical knowledge gap with respect to aquatic habitat restoration and management and is an interesting read overall. If the authors can conduct major revisions on the structure of their stage-structured population model and their justifications for using the parameter values and model scenarios that they did, I think this article will be suitable for publication in *PLOS One*.

Thank you for the helpful suggestions. We addressed them specifically in each comment below.

**Abstract**

1. Lines 5-7: My suggestion would be to delete this sentence, as the theme of this paper is specifically centered on salmonid habitat loss/creation.

*We edited the sentence to reflect that better.*

2. example of how you might re-phrase this sentence is to say you “used a stage-structured population model where each life stage was evaluated individually and as a component of the full model.”

*Thank you for the suggestion; we changed “in combination” to “simultaneously” to improve clarity.*

3. Line 17: Is widespread mortality at multiple life stages typical for coho salmon? Doesn’t it depend on the type of development action being enacted? How do mortality rates differ among life stages? I think there is room for a brief explanation of why coho salmon are an exemplary case study species (see below).

*We simulated a range of mortality rates for all life stages to cover many possible causes, and the wide range of values reflects the large uncertainty in how much mortality is caused by any given (or category of) development action. We argue that most development actions are likely to affect more than one life stage, through a variety of mechanisms, including both direct and indirect effects. We expand on the uncertainty around estimation of mortality rates and consequences for population models in the discussion (lines 313-319).*

*We expand on why coho salmon are good species for study in comment 7 below.*

4. Lines 18-19: I had trouble deciphering this sentence (specifically: “than if we assumed mean productivity or as compared to the typical size built in the PNW”). Please clarify.

*Thank you for the suggestion, we simplified the sentence to increase its clarity.*

**Introduction**

The introduction section is generally well formulated but would benefit from some changes to organizational structure and content.

1) I recommend shortening and combining the first two paragraphs of the introduction section and adding more history about the use of mitigation actions specifically for salmon habitat. I understand the impetus for starting with such broad subject matter — the readers of PLOS ONE have varied backgrounds outside of salmon ecology. That being said, many of them will be less interested in the jargon and more interested in the authors’ justification for conducting such a study.
2) I understand that theoretical approaches do not always fall into the “hypothesis-based science” category, but it would be nice to see more justification for your approach. The last paragraph would be strengthened if gaps in knowledge, study objectives, and predictions were explicitly listed out.

*Thank you for the suggestion. We added several sentences to the introduction to clarify gaps in knowledge, study objectives and predictions (lines 65-83).*

3) Readers would benefit from more information about coho salmon life history strategies and why they are an exemplary species for evaluating the efficacy of out-of-kind mitigation projects. Similarly, it’s unclear why river and stream restoration projects are considered “out-of-kind” to begin with. More explanation about this would be beneficial.

*We hope that the re-writing of the introduction based on comments 5 and 6 also addressed these concerns and clarified that river and stream restoration is more often done in “like-for-like” schemes, whereas “out-of-kind” offsetting is rather an exemption.*

*We also explain why we think coho salmon are good species for our modeling exercise in lines 67-69.*

8. Line 37 and throughout: Please move references to the end of the sentence whenever possible.

*We adjusted the sentence accordingly and revised the manuscript to adjust other sentences when needed, though references remained in the middle of sentences when needed for clarity.*

9. Line 41-42, 83, and throughout: I find that the way the terms “offsetting,” or “offsetting activities,” or “offsetting habitat,” are used to read awkwardly. Is offsetting habitat a policy term? If not, I would consider changing to “compensatory habitat” instead.

*We agree that the term “offsetting habitat” may be awkward, and changed it to “offsetting” throughout the manuscript to reflect practices in USA and Canada better.*

10. Line 45: Please change to “compensating and offsetting activities.” Also clarify, are the shortcomings characteristics of offsetting and offsetting themselves or the metrics used to evaluate them? Both?

*This sentence was erased when editing the introduction to improve focus.*

11. Lines 58-59 and throughout: Please reconsider the use of passive voice throughout your document. Using the active voice is often a more effective way of communicating your science and tends to read more easily. e.g., “developers proposed to compensate for fish entrainment by removing a dam that was 50-kilometers from the offending nuclear powerplant.”

*Thank you for the reminder, we checked the text throughout and edited accordingly where needed (e.g. lines 30-33, 38-45, etc)*

12. Lines 60-62: Please clarify. Were the restoration efforts themselves examples of out-of-kind offsetting or was it the effort to run the population model?

*Thank you to the reviewer for pointing out the awkwardness of the sentence; we split the long sentence in two and clarified that we meant that the restoration efforts themselves were example of out-of-kind offsetting.*

13. Lines 75-79: What about agricultural and urban development?

*We agree about the importance of agricultural and urban development as a threat to salmonids and added a citation to that effect (line 63), but do not specifically address those impacts with this model.*

14. Line 82: It would seem that restoration is a very specific example of out-of-kind offsetting. How does this exemplify this strategy as a whole? I would like to see more justification for 1) why river and stream restorations are examples of out-of-kind compensatory actions. It would seem to me that a lot of time restoration actions occur in areas directly adjacent to habitat loss or mortality-causing structures, and 2) why coho salmon are an optimal species to use in this kind of theoretical case study (see #3 above).

*We apologize for the confusion. We mention in lines 36-40 that most restoration actions are in fact “like-for-like” schemes, not “out-of-kind” schemes. In fact, examples of “out-of-kind” schemes are rather rare, and that is why we believe our modelling was interesting. We tried to make that clearer in lines 46-48. We also hope that the numerous edits made to the introduction to address comments 5, 6, and 7 clarified our approach and why coho salmon represent a good case-study to evaluate efficacy of “out-of-kind” offsetting.*

**Methods**

15. I haven’t worked with stage-structured population models in years, so I won’t speak to whether or not the authors have used them correctly here. What I would like to see is a table outlining each of the model simulations (e.g., egg mortality, fry mortality, adult mortality, multiple sources of mortality) and which parameters in the model were manipulated. I think readers would also appreciate an explicit ecological justification (with references) as to why and how certain parameters were manipulated for each simulation.

*We apologize for the confusion; Table 1 contains the information mentioned by the reviewer. The only parameter that is manipulated among scenarios is the extra (anthropogenic) chronic mortality; we added a sentence in the caption of Table 1 to emphasize that fact. Scenarios are differentiated based on when the extra chronic mortality φ_dist_ is applied (before density-dependent survival for the Egg scenarios, after density-dependent survival for the Parr scenarios, and in Year 3 for the smolt/adult scenario, e.g. after the smolts contributed by the offsetting channels are added to the main population; lines 174-178). Two parameters varied for each simulation: φ_dist_ and N_s_compH,_ which corresponds to the number of smolts contributed by the constructed side-channels. Justification for the range in values we used in the simulations for these two parameters are given in lines 115-119 and 172-174 (Fig 1) of the methods.*

16. What does “productivity” mean in this context? Does it refer specifically to the additional number of eggs and fry produced each year due to restored and created spawning habitat, or does it also include potential benefits to juvenile salmon growth and survival in response to added prey resources? If it does *not* include the latter, I think this is a major shortcoming in the described analysis — that restored and created habitats contribute to reproductive productivity and little else. The model likely underestimates survival rates and population growth, as added habitat can bolster productivity not only by adding physical space for redds, but also nursery habitat with terrestrial and aquatic prey subsidies and shelter from predation*. I would like to see an effort to include these habitat benefits in the model, or at the very least in a detailed paragraph in the discussion section.* For example, you could modify the carrying capacity (*k*) value in the Beverton-Holt density dependence function based on the amount of simulated compensatory habitat.

*We took productivity to refer to the additional number of smolts produced by offsetting habitat, i.e. it does include potential benefits to eggs, fry, and juvenile salmon growth and survival, and encompasses positive influence of added space for reproduction as well as nursery habitat and overwintering habitat. In other words, we do not differentiate the benefits from specific types of habitat but consider that the constructed side-channels provide all these benefits over time and we consider the cumulative benefits of the offsetting habitats in terms of “final output”, i.e. the number of smolts that enter the main population. We made that clearer in the methods when needed (e.g. line 184).*

*Since we consider that the constructed side-channels are always fully seeded in our simulations, by increasing the size of offsetting habitat, we effectively increase k of the overall river system. We expand on that aspect of our modeling further in comment 31 below.*

17. Lines 98-101: For clarity, I would rewrite this sentence as “To evaluate the amount of compensatory habitat required to maintain productivity for coho salmon populations experiencing chronic mortality, we modelled the offsetting potential of constructed habitats for salmon eggs, parr, and smolts/adults.”

*Thank you for the recommendation, we edited the sentence accordingly.*

18. Line 99 and throughout: Note that “salmon” should always come after “coho.”

*Thank you for that observation, we edited the text accordingly.*

19. Lines 107-112: Please move this information to the last paragraph of the introduction section. The paragraph can then be started something like: “We used several model scenarios to…”

*Thank you for the suggestion.*

20. Line 121: I’m surprised about the grouping between smolts and adults, as they experience very different drivers of mortality during the smoltification/out-migration phase and upstream migration/return phase. Either the model should be structured such that smolts and adults are two separate life stages in the matrix, or the authors should provide better justification as to why these very distinct life stages are grouped together.

*We agree that it is ecologically confusing to group both smolts and adults in the same stage. We did so because mathematically, in our model, we had no way of differentiating them since we did not have parameters to decompose smolt to adult mortality into discrete phases (like downstream migration, survival in estuary, mortality during first fall in the ocean, mortality during the last adult life in the ocean, and upstream migration). The only data available describe the mortality between smolting and spawning on redds. Therefore, any extra chronic mortality added after smolting in our models acts on the smolt-to-spawning adult survival phase as a whole. We tried to explain that better in lines 111-114.*

21. Line 162: Why was density dependence omitted from the baseline model? Isn’t this something that juvenile salmon encounter even if their habitat is pristine?

*Yes, that was a mistake, thank you for pointing it out.*

22. Line 162: I don’t see the initial population sizes listed anywhere.

*Good point – we added that information in the sentence.*

23. Line 168: This assumes that adult salmon are actively choosing to spawn in constructed side channel habitat with priority over main channel habitat. I worry about the validity of this assumption and I think it requires greater justification.

*We agree that this assumption is likely not totally realistic but don’t think that it would greatly affect the results of the simulations. It may over-estimate the contribution from the constructed side-channels by assuming that they are always fully seeded, in which case, our results under-estimate further the size of constructed side-channels needed. We added a sentence in the discussion (lines 283-285) to express that limitation of our model.*

24. Line 200: I would move this elsewhere so it’s not its own paragraph.

*Thank you, we did move it up.*

25. Line 210: Which citation was supposed to go here?

*We apologize for the missing citation and corrected the mistake*

**Results**

26. Line 216: Wasn’t this specifically the fry stage where you applied the Beverton-Holt function? Please clarify.

*Yes, it was. We clarified the sentence accordingly.*

27. Line 220: Was this supposed to be Fig. 2?

*Yes, sorry for the mistake. We corrected the sentence accordingly.*

28. Lines 232 and 234: No need to reference a figure multiple times in the same paragraph.

*Ok, we removed the reference to the figure*

29. Lines 237-246: It appears you’ve accidentally pasted the figure caption here.

*In our version and that sent by the journal, this was not the case.*

30. Line 256: Please see the comment above. It’s unclear what you mean by “productivity.” I’m also confused about whether your model assumes juvenile salmon stay in the same reach in which they are born. A fry produced in constructed side channel habitat will not stay there, but will move throughout the watershed until it migrates to the sea. Likewise, fry reared in the mainstem river will take advantage of constructed side channels periodically to feed and avoid predation. At what point are these two habitats considered separate and at what point are they combined in the model?

*We address how we define productivity in comment 16 above.*

*The model is not spatially explicit, so the two habitats are combined in the model.*

31. Line 262: The importance of the fry-smolt survival parameter further highlights the value of modifying the carrying capacity (k) value in the Beverton-Holt density dependence equation to account for the added side channel habitat.

*We agree that the model is sensitive to k, but that is not what we wanted to emphasize with our modelling and so that is why we fixed k at a set value. Since the offsetting habitat is seeded at k all the time, we effectively assume that it adds maximum production of smolt. In other words, increasing k would mean adding more habitat in our case, since k and the size of offsetting habitat are confounded and increase linearly. Therefore, instead of varying k for fixed sizes of offsetting habitat, we varied the sizes of offsetting habitat we modelled for fixed k (per m^2^). We explain that better in lines 163-165.*

**Discussion and Conclusions**

32. Lines 270-273: Just a reminder that your model was run specifically for coho salmon and this limitation needs to be elaborated on to begin with.

*We agree and edited the sentence to emphasize that fact.*

33. Line 279: See comments above. How is productivity defined here?

*Yes, see response to comment 16 above.*

34. Lines 284-288: I don’t see anywhere how you tested the effect of location on the magnitude of mitigation. I can see that you’re trying to substitute life stage for location here, but juvenile salmon can move throughout a watershed depending upon environmental conditions, prey availability, and ontogenetic cues, so the assumption that they would only occupy a habitat up or down stream of a created wetland needs to be further justified.

*Yes, our model is not spatially-explicit, and we addressed that limitation in our response to comment 30.*

35. Line 295: I’m glad you mentioned this an I think it’s worth expanding upon. Size is not everything when it comes to bolstering salmon populations. Adults and juveniles require appropriate habitat features to bolster their reproduction, growth, and survival, such as pebbly bottom substrate, large woody debris for shelter from predators, and overhanging vegetation to support prey inputs. An agency could build many hectares of stream habitat, but if it is not structurally and functionally useful then it may have little effect on salmon populations at all.

*Thank you for your supporting thoughts; we added a sentence in lines 289-291 to emphasize that point further.*

36. Lines 296-298: Please be wary of confusing productivity with habitat quality. Productivity refers specifically to the actual quantity of fish produced in terms of number of spawn or physical growth (in g/day). Also, what does “more demographically efficient” mean?

*Yes, we addressed the definition of productivity above in comment 16.*

*Re. “demographically efficient” (we assume you mean line 287): we agree the term was unclear and we changed it to “productive”.*

37. The Conclusions section should be framed in broader terms as in lines 377-382. Avoid using phrases like “our results” or “our analysis” and try to focus more on the big picture.

*We respectfully disagree with that comment and decided to keep our conclusions as is, i.e. as a summary of how our results fit within the broader literature.*

**Other**

Please consider making your model code publicly available on GitHub or a similar repository and linking to it in a Data Availability Statement.

*Thank you for the suggestion; we added an annotated example of the R code used to run the model in the Supplemental Material.*
